# Supplementary material for: DMRforPairs: identifying Differentially Methylated Regions between unique samples using array based methylation profiles
Source: BMC Bioinformatics. 2014 May 15;15:141. doi: 10.1186/1471-2105-15-141 (PMC4046028; doi:10.1186/1471-2105-15-141)
Supplement: Additional file 2 — DMRforPairs output for the comparison of A431-MCF7 and NA17018-NA17105. Please start from the HTML files in each folder. Available via the BMC Bioinformatics website. [file 1471-2105-15-141-S2.zip › 1394847754114233_MOESM2_ESM/A431_MCF7/figures/40012.pdf]

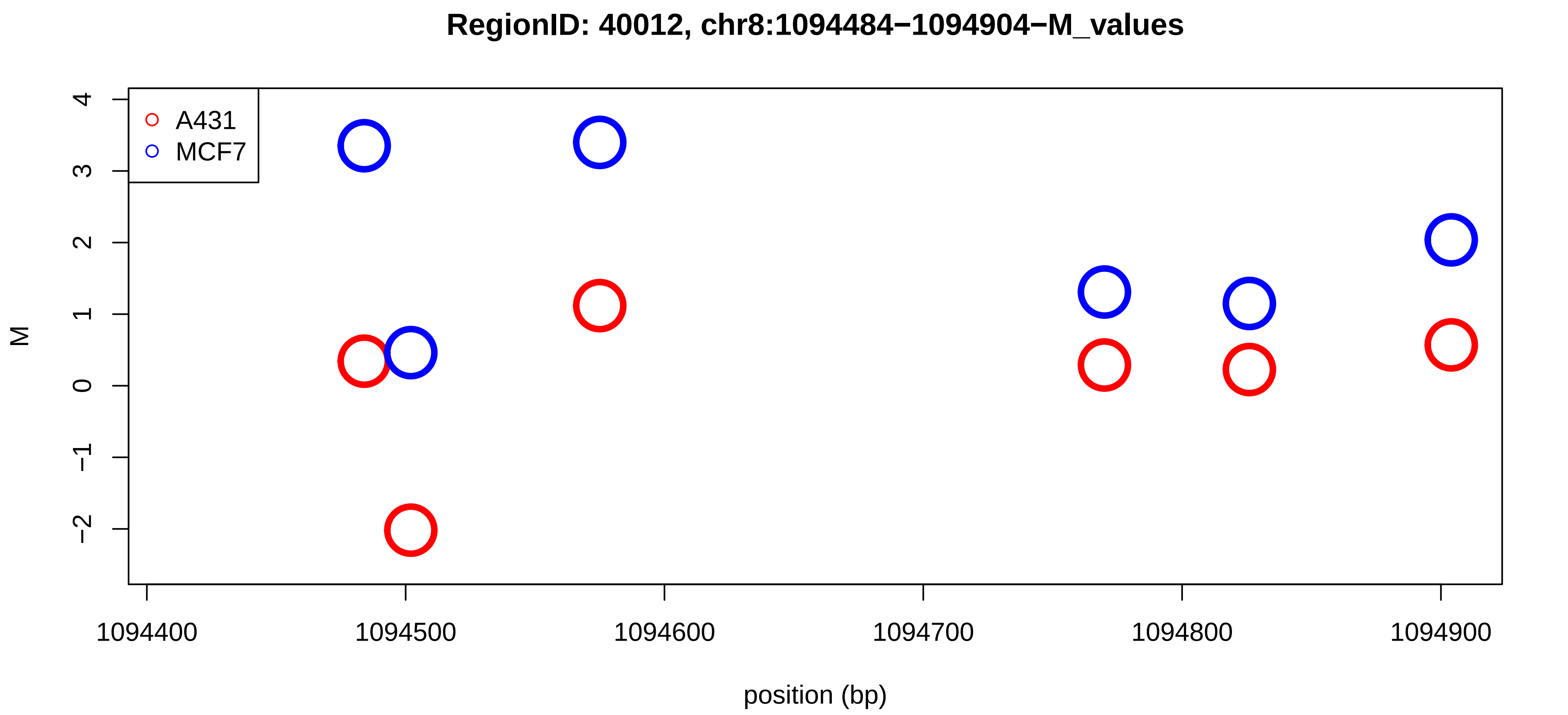
RegionID: 40012, chr8:1094484−1094904−M_values
A431
MCF7
M
position (bp)
1094400
1094500
1094600
1094700
1094800
1094900
−2
−1
0
1
2
3
4

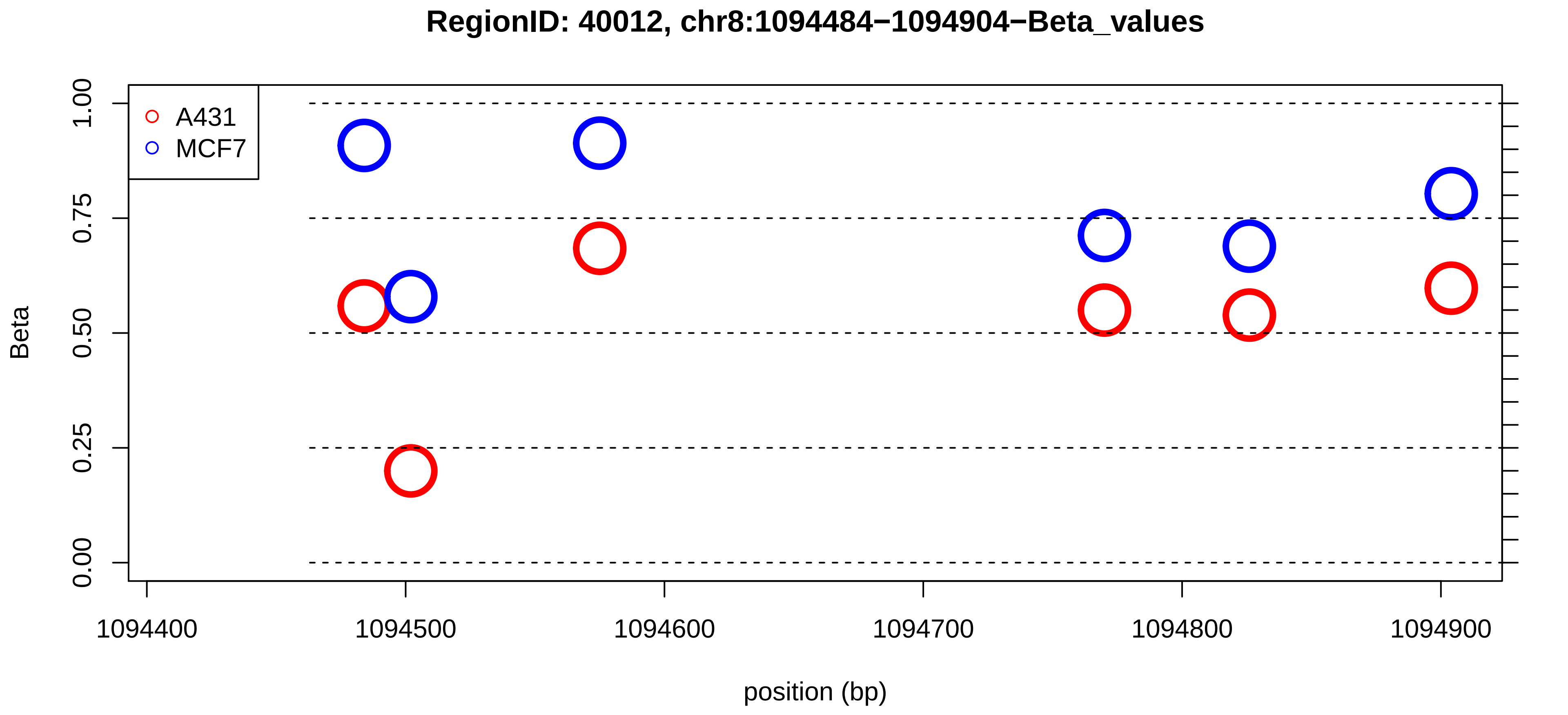
RegionID: 40012, chr8:1094484−1094904−Beta_values
A431
MCF7
Beta
position (bp)
1094400
1094500
1094600
1094700
1094800
1094900
0.00
0.25
0.50
0.75
1.00

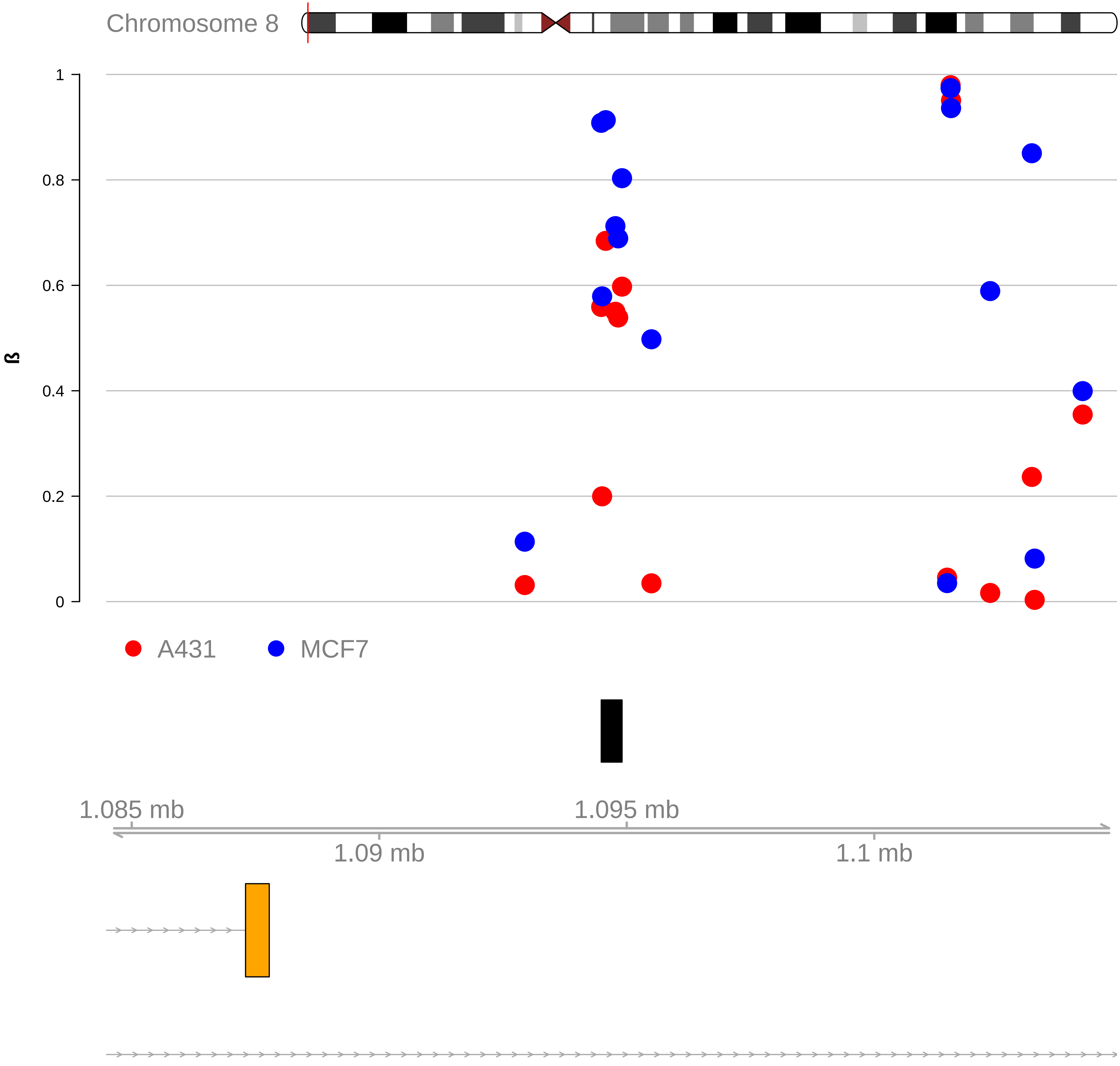

Chromosome 8
1
0.8
0.6
0.4
0.2
0
ß
A431
MCF7
1.085 mb
1.095 mb
1.09 mb
1.1 mb
